# On the beauty of vases: Birkhoff’s aesthetic measure versus Hogarth’s line of beauty

**DOI:** 10.3389/fpsyg.2023.1114793

**Published:** 2023-04-20

**Authors:** Ronald Hübner, Emily Sophie Ufken

**Affiliations:** Department of Psychology, University of Konstanz, Konstanz, Baden-Württemberg, Germany

**Keywords:** vases, beauty, curvature, Birkhoff, aesthetic measure, Hogarth, line of beauty

## Abstract

Vases continue to be important aesthetic objects in almost all developed cultures. Nevertheless, there is little to no systematic research on the shape characteristics that determine their beauty. A famous exception is Birkhoff, who in his 1933 book used the geometric ratios of vases to calculate their beauty. One form factor that he discussed theoretically but did not include in his aesthetic measure is the outline curvature of vases. This is despite the fact that William Hogarth recognized curvature as relevant to the aesthetic evaluation of forms as early as 1753, demonstrating this with his Line of Beauty. Given the great influence of these two ideas, the aim of the present study was to examine their contribution to the aesthetics of vases. For this objective, we designed a set of symbolic vases by systematically varying width and curvature, and asked participants to rate their beauty in an online experiment. The results show that both geometric ratios and curvature contribute to the beauty of the vases.

## 1. Introduction

Almost all developed cultures have a long history of the form and function of vases, and even vanished cultures left vases as an early testimony of their artistic creation (Cooper, 2000). Given the long history and the wide distribution of vases to the present day, it is astonishing that the shape characteristics determining their beauty have hardly been studied systematically. One rare exception is the American mathematician George David Birkhoff (1884–1944). In 1933 he published his book “Aesthetic Measure,” in which he introduced a quantitative definition of the concept *unity in variety*, one of the oldest universal aesthetic principles (Hutcheson, 1725; Fechner, 1876). This principle states that humans find objects beautiful when they have different parts (variety) that are related by some common feature or can otherwise be conceptualized as a coherent whole (unity). In his introspective approach, Birkhoff (1933) operationalized variety by the complexity *C* of an object and unity by its order *O*. He then defined an aesthetic measure *M* by the quotient *O* over *C*, i.e., *M* = *O*/*C*, and proposed that it predicts the aesthetic appreciation of that object. Birkhoff applied his measure to various types of objects, such as polygons, ornaments, tiles, and, most importantly for the present objective, vases.

Birkhoff demonstrated the validity of his measure by calculating *M* for several examples of each object type and stating that there is a good agreement with his self-assessed beauty. Unfortunately, later empirical studies found little support for the measure *M* (Davis, 1936; Beebe-Center and Pratt, 1937; Wilson, 1939; McWhinnie, 1968). This may not be surprising, since Birkhoff included only some of the relevant object variables in his measure, as he himself acknowledged. His focus was mainly on the geometric relationships between certain dimensions, and even that only to a limited extent. Other object features were largely neglected. With respect to the beauty vases, for instance, he discussed the important role of curvature, but did not integrate it directly into his measure.

Despite the limited success of his beauty measure *M*, Birkhoff’s approach has widely been appreciated as the first attempt of quantifying the aesthetic value of an object. Furthermore, it has an ongoing influence in various areas of aesthetics (Douchová, 2016). With respect to vases, for instance, Birkhoff’s measure was not only improved (Staudek, 1999), but also used in computer aided design to define a fitness function for selecting aesthetically pleasing vases by an evolutionary algorithm (e.g., Reed, 2013).

In the present study, we examined the role of curvature for the beauty of vases and its relation to Birkhoff’s aesthetic measure. For this objective we systematically varied the degree of curvature and the width of vases. However, before we report details of our procedure and the results, we briefly review relevant concepts and earlier results.

Birkhoff (1933) proposed that the beauty of vases depends on three factors: (1) the regularity of their outline, (2) their utilitarian and conventional requirements, and (3) the unique geometric relationships between the involved dimensions. His definition of *C* and of *O*, however, takes only the third factor into account. He admitted that the other two factors are also relevant but conceded that it is difficult to formulate them precisely. Given this restriction, he applied his measure only to eight Chinese vases, mainly because they were rotational symmetric. Moreover, he only considered their visual contour.

According to Birkhoff, the complexity of an object reflects the amount of perceptual attention necessary for its processing. With respect to vases, he assumed that the necessary attention depends on the number of *characteristic points* along the contour line. Such points are the end points of the contour, points whose tangent is vertical (e.g., at the minimum and maximum width), points where curvature changes its direction from concave to convex (inflection points), and corner points where the direction of the tangent changes abruptly. The sum of these points is taken as the value of complexity *C*. An example from our set of symbolic vase stimuli is shown in Table 1. For this vase there is a characteristic point per side at the minimum width, maximum width, inflection, top, and base. These 10 points lead to a complexity value of *C* = 2 × 5 = 10.

**Table 1:** Characteristic network for one (number 12) of our vase stimuli.

*C* = 2 × 5 = 10			*O* = *H* + *V* + *HV* = 5
*Points per side*	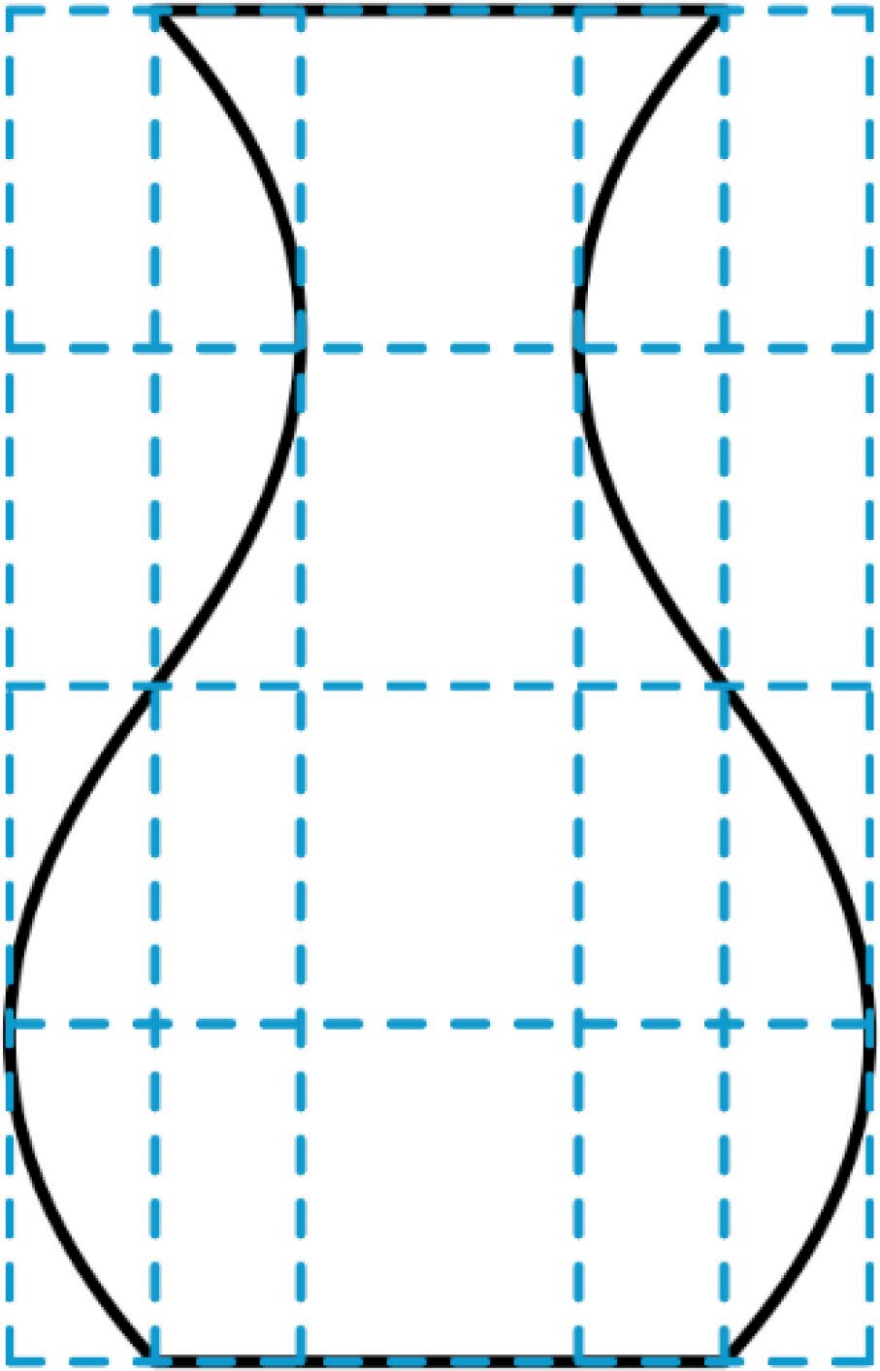		*Ratios*
1 top		1:1	base width/top width
		1:1	base width/inflection width
1 minimum			(other ratios are dependent)
		*H* =	2
1 inflection		1:1	base-to-max/max-to-inflection
		1:1	max-to-inflection/inflection-to-min
1 maximum		1:1	Inflection-to-min/min-to-top
			(other ratios are dependent)
1 base		*V* =	3
		*HV* =	0

On the basis of the characteristic points, a *characteristic network* of lines can be drawn, as shown in Table 1 for our example vase. The geometrical relations among the distances within this network are the relevant variables for defining the order *O*. For Birkhoff, the order of an object is the number of its harmonic relations and regularities. More specifically, *O* is defined by the number of cases in which the independent relationships between the distances in the characteristic network are in the ratio of 1:1 or 1:2. Birkhoff considered three types of relevant relations: (1) relations *H* between horizontal distances (maximal 4), (2) relations *V* between vertical distances (maximal 4), and (3) relations *HV* between horizontal and vertical distances (maximal 2). It should be noted that he also considered *characteristic tangents*. However, because they are irrelevant for our vase stimuli, order is defined in the present case by *O* = *H* + *V* + HV.

With respect to the horizonal distances, our stimuli are special as the base width, inflection width, and top width are equal for each vase. Accordingly, we have a 1:1 relation between the horizontal width of base and top, of base and inflection, and of inflection and top, which remain invariant to changes in curvature and width. Because only two of them are independent, and there are no other relevant horizontal ratios for our example vase, we have *H* = 2 in this case.

If we consider the vertical distances in our example, then those between base to maximum, maximum to inflection, inflection to minimum, and minimum to top are equal. Accordingly, there are three independent ratios of 1:1. Because there are no relevant ratios between horizontal and vertical distances, we finally have *O* = *H* + *V* + *HV* = 2 + 3 + 0 = 5. Thus, the aesthetic measure of the vase in Table 1 is *M* = *O*/*C* = 0.5.

As mentioned, Birkhoff also considered functional aspects. For him a vase is “…a useful container which should be of substantial capacity, stable in horizontal position, easy to handle, empty, and move about.” (p.75). From these requirements he derived several constraints. For instance, he demanded that the maximum width of a vase should be at least a quarter of its height, while its minimum width, which should be in the upper half of the vase should be at least one-eighth of its height. These and other demands imply, for instance, that the optimal ratio of minimum and maximum is 1:2.

A great weakness of Birkhoff’s measure *M* is that it only takes specific ratios of the distances into account. Accordingly, vases deviating only a small amount from the ideal ratios of 1:1 and 1:2 already have a lower measure. Moreover, vases that deviate from the ideal to varying degrees are given the same value. That this leads to a poor differentiation between vases has been demonstrated by Staudek (1999). He designed 15 symbolic vases by morphing the outline of a wide vase step by step into a small one. As a result, their aesthetic measure comprised only five values. Moreover, each of the two most frequent values alone represented six vases. Therefore, Staudek (1999) extended Birkhoff’s measure by introducing a tolerance margin in which the value is proportionally reduced with the difference between the actual and the desired ratio. Consequently, vases deviating only little from the ideal have a higher order value than vases deviating more. We will refer to this extended aesthetic measure as *M*_e_.

As mentioned, Birkhoff (1933) admitted that the beauty of vases not only depends on the geometric relations, but also, among others, on the regularity of their outline. Although this property is purely formal, Birkhoff remarked that it is rather difficult to formulate precisely, especially, if the outline is curvilinear, what is frequently the case. He argued that curves, unlike straight lines or circles, are infinitely complex, because there is no finite set of points that determines the curves completely. This seems to be one of the reasons why he did not include outline curvature in his aesthetic measure. Instead, he used the characteristic points of vases and some qualitative properties to roughly assess the regularity of curved outlines. For an outline to be considered as regular, Birkhoff demanded that its curvature between the characteristic points should vary continuously with a rate of change as small as possible. Moreover, curvature should not oscillate more than once between the points of inflection. Birkhoff (1933) concluded that “[t]he eye can follow with ease curves meeting these two requirements, just because of the small curvature and its small rate of change” (p. 74). Today one would say that the curves should be “fair” (Burchhard et al., 1994). Furthermore, it is nowadays also possible to completely determine and describe large classes of curves by a limited number of parameters of, for instance, Bézier curves.

**Figure 1:**
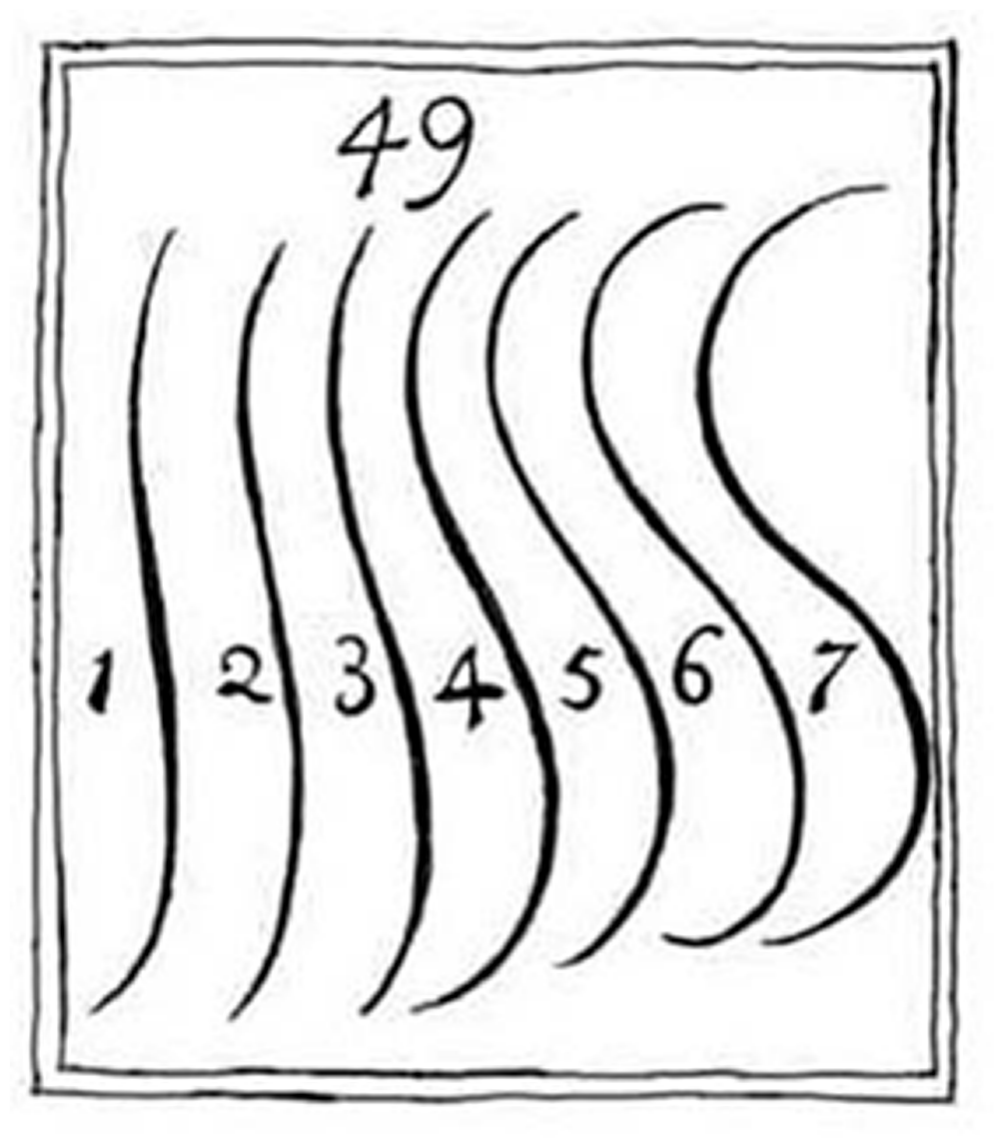
Illustration 49 from Plate I in the book “The Analysis of Beauty” (Hogarth, 1753) (Copy downloaded from: https://commons.wikimedia.org/wiki/File:Serpentine_lines_from_William_Hogarth%27s_The_Analysis_of_Beauty.jpg).

It is remarkable that although Birkhoff (1933) devoted much space to the discussion of curvature and applied his measure to Chinese vases with a serpentine contour, he did not mention the ideas of the British artist and theorist William Hogarth (1697–1764) on curvature. In his book from 1753 Hogarth not only proposed that serpentine lines are more beautiful than straight or simple curved lines, but also presented seven S-shaped lines (see Figure 1) and declared line number 4, which he called “Line of Beauty,” the most beautiful. Over the years there was widespread agreement that serpentine lines, often generally referred to as *line of beauty*, are beautiful. However, Hogarth’s claim that one curve is the most beautiful was largely rejected. Wundt (1874/1903), for instance, wrote: “…the attempt to find an absolute curve of beauty, as made by Hogarth (1753), for example, is mistaken, since the degree and form of the pleasant curvatures depend on the other properties of the objects.” (p. 151, translated by R.H.).

For contour lines it might be true that their preferred degree and form of curvature depend on other properties of the object. Nevertheless, it might also be worthwhile to consider pure serpentine lines and investigate the degree of curvature that is most liked. However, although a large number of studies have shown that curved shapes are, on average, preferred over angular ones (for overviews see Bertamini and Palumbo, 2015; Corradi and Munar, 2020), the preference of specific curvatures has rarely been examined. Before 2022, no researchers had even empirically tested Hogarth’s claim concerning his Line of Beauty. Hübner and Ufken (2022) were the first in this respect. They found that line number 4 (i.e., the Line of Beauty) was indeed perceived to be very attractive but was not seen as more beautiful than line number 5. Furthermore, the relationship between mean absolute curvature and preference was well described by an inverted U-shape. A recent study even provided evidence that the preference for Hogarth’s Line of Beauty is an evolutionary by-product (Hübner et al., 2023).

## 2. Experiment

In the present experiment we wanted to investigate both, the role of geometric relationships and of outline curvature for the beauty of vases. According to Birkhoff (1933), certain geometric ratios are crucial, while Hogarth would assume that a specific curvature is essential. Different from these authors, however, we assumed that there are not *all or nothing* relations between values of the relevant variables and beauty, but that the relations are continuous. Moreover, we assumed that the optimal values for beauty vary across conditions. For investigating these issues, we systematically varied the outline curvature and width of symbolic vases. We have chosen a range of curvature that is likely to have an inverted U-shaped relationship with beauty. For the selected range of width, we expected the same.

A problem for investigating the effects of geometric ratios and curvature on the beauty of vases is that both variables are not independent from each other. In the present case this especially holds for the minimum width to maximum width ratio (MMR), which largely varies with outline curvature. The larger the curvature the smaller the MMR. However, the MMR also depends on the width of the vases. Thus, the reduction in MMR due to an increase in curvature can at least partially compensated for by an increase in width.

We hoped that it would be possible to separate the effects of the different variables using multiple regression. It was also of interest to see how close the optimal MMR will be to 1:2, and the optimal outline curvature to that of Hogarth’s Line of Beauty. For comparison, we also considered the measures *M* and *M*_e_. Finally, to assess how the preferred curvature of single lines differs from that of vase outlines, we asked our participants not only for the assessment of the beauty of symbolic vases, but also of corresponding isolated S-shaped lines.

### 2.1. Methods

#### 2.1.1. Participants

Sixty participants (mean age 23.5 years, SD = 3.44, 20 male), most of which were university students, were recruited for participation in the online study. For completing the study, participants received an Amazon voucher worth € 3. The experiment was conducted in accordance with the ethical guidelines of the University of Konstanz and the Declaration of Helsinki (1964) and its later amendments. Participants were informed of their right to quit the study at any time without reprisal and their informed consent was obtained by check-marking a box before the actual experiment started.

#### 2.1.2. Stimuli

**Figure 2:**
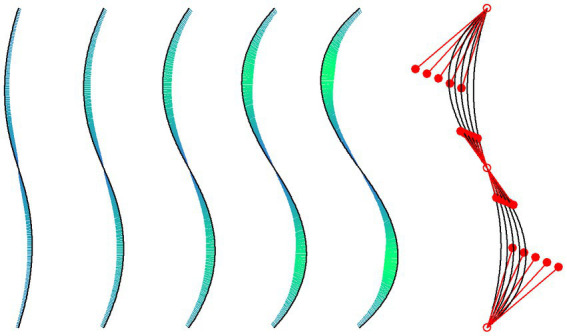
The five isolated lines represent the right vertical contour lines of our vase stimuli. The curvature at points along the lines is indicated by the length (scaled for good visibility) and color (redundant coding) of the short lines orthogonal to the tangent at these points. On the right, the five lines are superposed and shown together with their corresponding Bézier control points (empty red dots) and handle points (filled red dots).

The symbolic vase stimuli were constructed from five S-shaped lines, each of which consisted of two cubic Bézier curves (see Figure 2). A cubic Bézier curve is defined by the *x* and *y* coordinates of four control points. The first and last control points are the start and end points of the line, respectively, while the other two control points, also called *handle points*, determine the curvature of the line. In our case an S-shaped line was constructed by defining the endpoint of an upper Bézier curve as the starting point of a lower curve (see Figure 2). Because the height of each component curve was 150 px, the overall stimulus height was 300 px. Starting from a line with a low curvature, the other four lines were obtained by increasing curvature. For this objective, the outer handle points were systematically moved outwards (see Figure 2). The first handle point of the upper component line for the least curved line had coordinates *x* = −30 and *y* = −75, relative to the top starting point. The corresponding coordinates for the other lines are *x* = (−43, −57, −70, −84), and y = (−70, −66, −61, −57). The coordinates were chosen in such a way that the distance between the horizontal minima (and maxima) was ~8 px from one line to the next (see Figure 2). Because the point where the two component lines meet, is also the inflection point, i.e., the point where curvature changes polarity, a smooth transition of curvature between the two components was achieved by choosing the coordinates of the corresponding handle points in such a way that the curvature at the connection point was identical for both component lines. The curvature at each point of the individual lines can be seen for the separated lines in Figure 2. The lines and their curvature were computed with functions from R package “knotR 1.0–2” (Hankin, 2017). The mean absolute curvatures of the five lines are: 0.00483, 0.00640, 0.00766, 0.00865, and 0.00941. At some places, we will simply denote these curvatures by numbers from 1 to 5, respectively.

To assess how similar our individual lines are to Hogarth’s lines, we computed the RMSD (root-mean-square deviation) between our five lines and Hogarth’s lines. These measures revealed that the numerically most preferred line 3 is most similar to Hogarth’s line 5 (RMSD = 26.1). Lines 2 and 4 are most similar to Hogarth’s lines 3 (RMSD = 26.3) and 5 (RMSD = 19.8), respectively. If we compare Hogarth’s line number 4, i.e., his Line of Beauty, with our five lines, then it is most similar to line number 2 (RMSD = 39.2).

Each line and its mirrored counterpart were used as vertical contour lines for constructing our symbolic vase stimuli. For creating the horizontal contours, i.e., the base and top of his vases, Birkhoff (1933) used a perspective projection, which resulted in convex elliptical lines. In the present study, we plotted the cross-section of the vases, i.e., we simply connected the two start points and the two end points by straight lines (for an example see Table 1). For each vertical contour line, five vases were created by systematically increasing the width at the base and top from 95 to 195 px in steps of 25 px. The resulting 25 vase stimuli, which can be seen in the Results section, are relatively simple. They represent vases that are rotationally symmetric. Moreover, the vertical contour lines were centrally symmetric, i.e., they are invariant under point reflection (rotation of 180 degree) through its center. Consequently, the width at the base, inflection, and top are the same.

**Figure 3:**
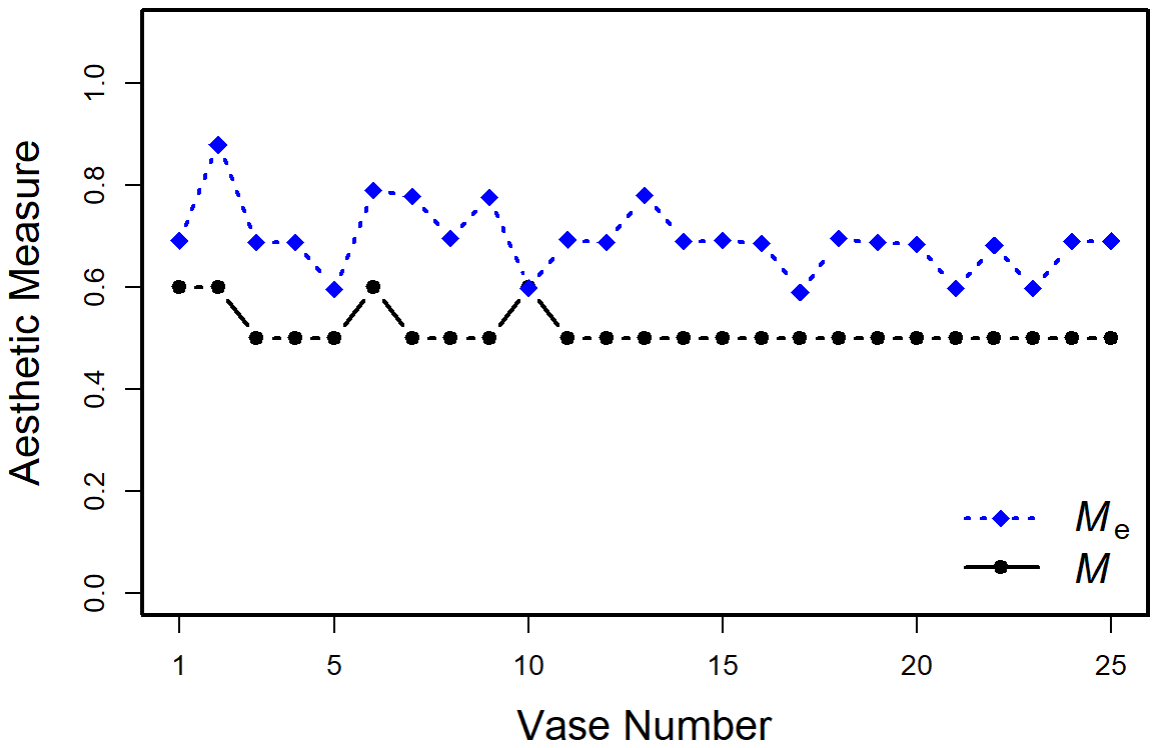
Birkhoff’s aesthetic measure *M* and Staudek’s extended version *M*_e_ applied to our 25 vase stimuli.

We also computed the aesthetic measures *M* and *M*_e_ for our 25 vases. Because, due to rounding errors, the computations of *M* are somewhat unprecise, we applied a small tolerance margin of 1% for categorizing a ratio as 1:1 or 1:2. Nevertheless, as can be seen in Figure 3, *M* is the same for most of the stimuli, because it only produces two different values. The variation is somewhat larger for *M*_e_, where a tolerance margin of 10% was used.

#### 2.1.3. Procedure

The experiment was conducted as first part of a larger online study. The results of the other parts will be reported elsewhere. The program was written in Javascript. At the beginning participants were shortly introduced to the topic and procedure of the study. After consent and providing personal information (gender, age), a specific instruction for the experiment’s task was presented. To achieve standardized visual quality of stimuli presentation, participants were informed that they had to use a computer. The program stopped if a mobile device was used. All stimuli were presented in black on a white 500 × 500 px square on the screen.

The experiment consisted of two phases. In the first phase the participants had to rate each of the randomly presented vase stimuli according to how much they liked them (from “I do not like it” to “I like it very much”), on a visual analog scale internally ranging from 1 to 100. We consider these ratings as beauty ratings.

In the second part, which also started with a specific instruction, the five lines (Figure 2) were separately presented in randomized order and had to be rated in the same manner as the vases. At the end, participants could enter their e-mail addresses for receiving the voucher or select the option to anonymously donate their data. Altogether, the experiment lasted about 10 min.

### 2.2. Results

**Figure 4:**
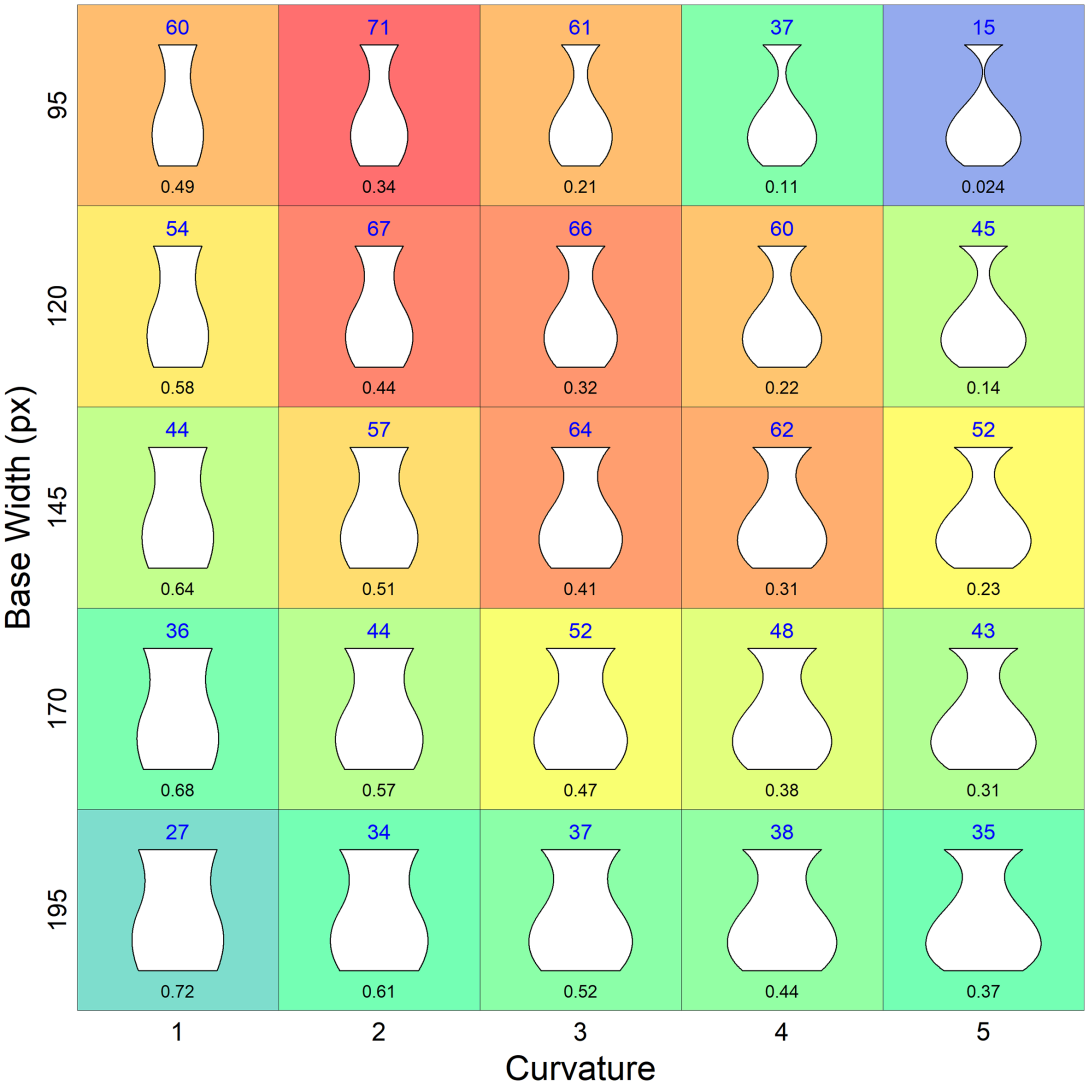
Mean beauty ratings of the vases. The values are provided as number above the vases as well as the color of a heat map (redundant coding). The numbers under the vases indicate the MMR.

The mean ratings of the vases, which range from 15 to 71, are shown in Figure 4 both as numbers and colors of a heat map.

#### 2.2.1. Interindividual variability

To examine the degree to which the aesthetic preferences were shared across participants, we computed the across-participant average MM1 (mean-minus-one) correlation measure (Vessel et al., 2018). That is, we first computed the correlation between each participant’s ratings and the average of all other participants. The MM1 score is then the mean of these correlations. In our case, we obtained an MM1 score of 0.64, which indicates that the common preference across participants was relatively high. This result confirms those showing that the aesthetic appreciation of form is less individual than that of content and artworks (Chatterjee, 2004; Vessel et al., 2018).

#### 2.2.2. Regression models

Given the high level of agreement among the participants with regard to their beauty ratings, we analyzed the data by means of traditional multiple-regression models. First, we used the mean absolute curvature (often simply called “curvature”) of the contour lines and the base width (often simply called “width”) as predictors, where both variables were entered as a quadratic polynomial into the regression. As a result, the model accounts for 96% of the variance of the mean ratings, *F*(8, 16) = 51.6, *p* < 0.001, *R*^2^ = 0.96. As can be seen in Table 2, the linear as well as the quadratic components of the two polynomials were significant. This confirms the assumed inverse U-shaped relationship with beauty for the two variables. However, of the four potential interactions, three were also significant. Both components of the curvature polynomial interacted with the linear component of the width polynomial. In addition, the linear component of the curvature polynomial also interacted with the quadratic component of the width. The latter interaction reflects the relatively strong reduction of the mean ratings for vases of small width when curvature is large.

**Table 2:** Result of the multiple regression analysis with quadratic polynomials of curvature (C) and width (W) as predictors.

Coefficient	Estimate	Std. Error	*t*-value	Value of *p*
Intercept	48.4	0.663	73.0	<0.001***
C	−8.3	3.32	−2.52	0.02*
C^2^	−32.0	3.32	−9.64	<0.001***
W	−30.4	3.32	−9.17	<0.001***
W^2^	−29.1	3.32	−8.77	<0.001***
C × W	156	16.6	9.44	<0.001***
C^2^ × W	83.5	16.6	5.03	<0.001***
C × W^2^	−101	16.6	−6.11	<0.001***
C^2^ × W^2^	17.9	16.6	1.08	0.296

The corresponding formular for the R program “lm” is: Beauty ~ poly(C,2)*poly(W,2).

Because curvature and width determine the ratio between minimum and maximum width, it seemed that the MMR could represent the interactions. Indeed, when we entered curvature, MMR, and width into a regression without interaction terms, then it also accounted for 96% of the variance (see Table 3), *F*(8, 16) = 76.1, *p* < 0.001, *R*^2^ = 0.96.

**Table 3:** Result of the multiple regression analysis with quadratic polynomials of curvature (C), MMR, and width (W) as predictors without interaction terms.

Coefficient	Estimate	Std. Error	*t*-value	Value of *p*
Intercept	48.4	0.630	76.8	<0.001***
C	−434	91.9	−4.72	<0.001***
C^2^	−8.11	3.65	−2.22	0.039*
W	252	63.4	3.97	<0.001***
W^2^	−46.6	7.31	−6.38	<0.001***
MMR	−504	111	−4.43	<0.001***
MMR^2^	−96.6	11.6	−8.30	<0.001***

The corresponding formular for the R program “lm” is: Beauty ~ poly(C,2) + poly(W,2) + poly(MMR,2).

**Figure 5:**
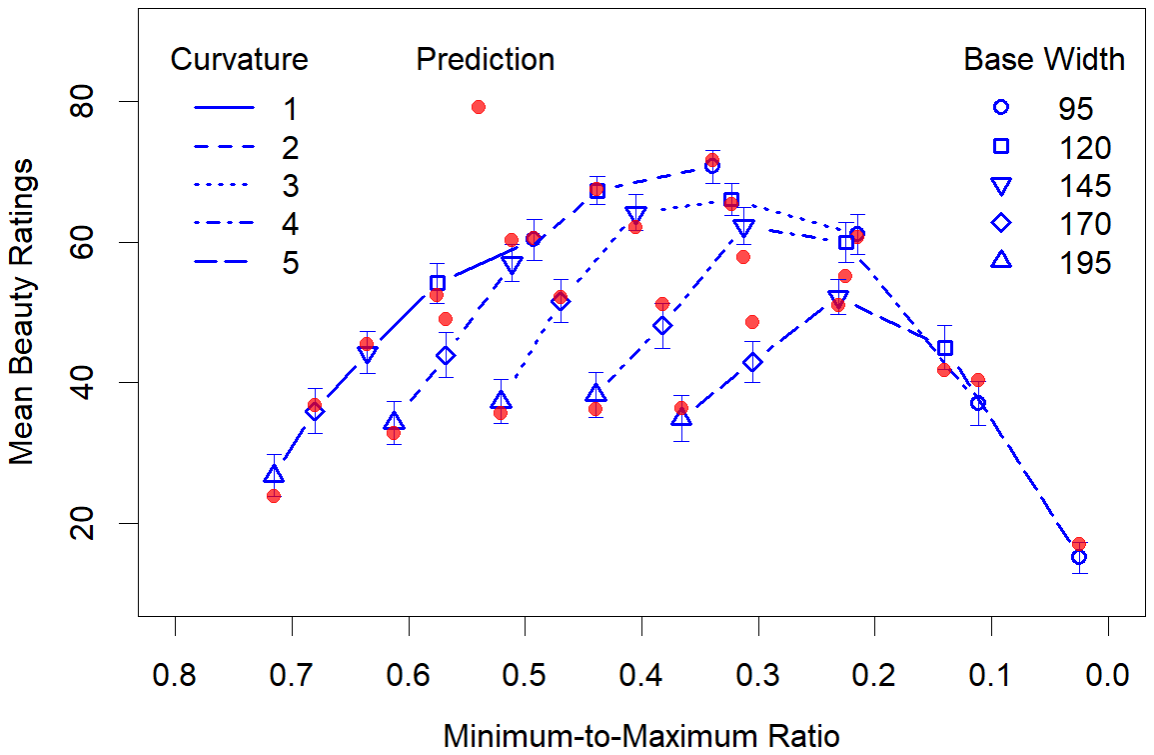
Mean beauty ratings as function of the MMR for the different widths and curvatures. The red filled dots represent the prediction by the second regression model (see details in the text). Note that the values of the X-Axis decrease from left to right to make their direction compatible with that in Figure 4.

The result of the latter regression supports the idea that the interactions between curvature and width in the former regression reflect the effect of the MMR, which simplifies the interpretation of the results. Moreover, if we plot the mean ratings as a function of the MMR, then the curves for the different base widths are somewhat shifted apart along the X-axis (Figure 5), compared to using curvature on the X-axis (similar as in Figure 4), which makes the effects of the variables more clearly visible. First of all, by inspecting Figure 5 it becomes clear that there is an overall inverted U-shaped relationship between MMR and the mean beauty ratings. Furthermore, it can be seen that this relation is largely due to the fact that the beauty of vases with smaller curvature increases with width, whereas for vases with a larger curvature this relation changes in the direction of an inverted U-shape, meaning that a smaller width decreases the beauty of the vases. Obviously, this decrease is mainly driven by a suboptimal range of the MMR. Despite these relatively complex relations, the regression model predicts the data quite well, as can also be seen in Figure 5.

#### 2.2.3. Testing the contribution of curvature

Obviously, the MMR resulting from our selection of widths and curvatures produced a large variance in the beauty ratings. Therefore, the question was to what extent curvature also produced some effect independent of its contribution to the MMR. Unfortunately, we cannot directly interpret the significance of the coefficients in the second regression, because combining curvature and width in the MMR produced multicollinearity. Indeed, while the VIF (variance inflation factor) was 1 for all variables in the first regression, indicating that there was no multicollinearity (Frost, 2020), it was 1,120, 445, and 1858 for curvature, width, and MMR, respectively, in the second regression. However, although multicollinearity is a problem for interpreting the value of the parameters and trusting their significance, it is no problem for prediction (Frost, 2020). Therefore, we tested whether curvature also had a direct effect on the ratings by stepwise regression. First, we used the polynomials of width and MMR as predictors in the regression, which revealed an *R*^2^ of 0.90. Then we added the polynomial of curvature as further predictor, which significantly increased *R*^2^ to 0.96, *F*(18, 2) = 15.3, *p* < 0.001. Thus, we can conclude that curvature had a significant effect on the ratings, independent of its indirect effect through the MMR.

#### 2.2.4. Aesthetic measures

To examine how well Birkhoff’s measure accounts for the vase ratings, we correlated the *M* scores with the mean beauty ratings. The correlation was *r* = 0.20, *t*(23) = 1.00, *p* = 0.327. When we did the same with the *M_e_* scores, we obtained *r* = 0.48, *t*(23) = 2.64, *p* < 0.05. This demonstrates that introducing a tolerance margin improves the predictive power of *M*, although still only 23% of the variance are explained.

#### 2.2.5. Isolated lines

**Figure 6:**
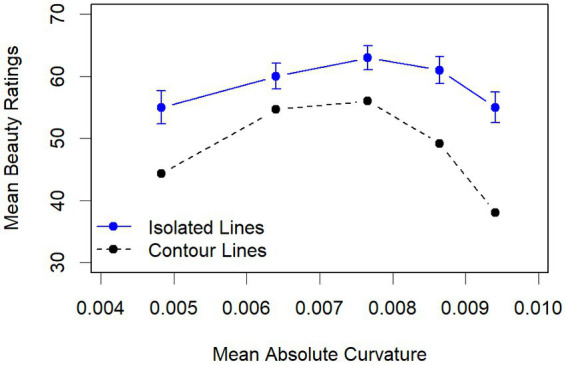
Mean beauty ratings of the isolated lines and mean beauty ratings of the contour lines across vase width as function of their mean absolute curvature. The error bars indicate the standard error.

The mean ratings of the isolated lines are shown in Figure 6. As can be seen, their range is relatively small (55–63). Nevertheless, a statistical analysis confirmed that there is a quadratic relation with the mean absolute curvature. Because there were only five data points for each participant, and the interindividual variability in the ratings was relatively high (MM1 score of 0.26), we computed a linear random mixed model across all data with participants as random factor and a quadratic polynomial of mean absolute curvature as predictor. It revealed that of the three coefficients (Intercept: 59.0; C: 8.13; C2: −50.4), in addition to the intercept, only the quadratic component was significant, *t*(59) = −4.07, *p* < 0.001. Numerically, line 3 was most preferred. However, Bonferroni-adjusted pairwise *t*-tests revealed that only the mean rating of line 1, *t*(59) = 2.98, *p* < 0.05, and of line 5, *t*(59) = 2.98, *p* < 0.05, deviate significantly from that of line 3.

For comparison, we also plotted the mean beauty ratings of the vase stimuli with the corresponding contour lines across vase width in Figure 6. As can be seen, not only do the ratings again show the inverted U-shaped relationship between mean absolute curvature and beauty, but also that this relationship is quite similar to that of the isolated lines. The mean ratings also increased with an increased curvature across width for smaller curvature, reached its peak for curvature 3, and decreased for larger ones. The decrease was more pronounced than that of the ratings of the isolated lines, which reflects the amplification of the negative effects of larger curvature through the corresponding MMRs, especially for small vases.

## 3. Discussion

The results of our study show that both geometric relationships, as proposed by Birkhoff (1933), and curvature, as emphasized by Hogarth (1753), contribute to the beauty of vases. Our set of symbolic vases was designed by systematically varying width and curvature over five values each, resulting in 25 stimuli. Based on our former study (Hübner and Ufken, 2022), we expected that curvature has an inverted U-shaped relation with beauty, meaning that lower and higher values are less liked than medium ones. A similar relationship was assumed for the width of the vases. Accordingly, we analyzed the data by using quadratic polynomials of these variables and all their possible interactions as predictors in a multiple regression, which accounts for an astonishing 96% of the variance in the mean beauty ratings. However, our results also show that there were complex interactions between width and curvature. Because both variables strongly affected the MMR, it was reasonable to assume that the interactions reflect the effects of this ratio. Indeed, if we replace the interaction terms with the MMR, we find that the same high percentage of variance can be explained. Moreover, this assumption is helpful in understanding our data pattern. If we consider Figure 5, in which the MMR is used as quantity for the abscissa, then we see that the beauty ratings of the vases with small curvature monotonically increased with decreasing width. However, starting with vases of medium curvature, the ratings only increased for the broader vases, but decreased for smaller ones. Furthermore, the greater the curvature, the earlier and the more pronounced this decrease was. Thus, there was a modulation of the functional relationships between curvature, width, and beauty by the MMR. Specifically, with an increasing curvature the relationship between width and beauty transforms from an almost linear to an inverted U-shaped function, because more and more of the smaller vases have an MMR that was not liked.

In contrast to our continuous approach, Birkhoff’s (1933) measure *M* had almost no predictive power. As we have seen in Figure 3, the measurement produced only two different values for our set of vases, which is even less than the five values for Staudek’s (1999) vases. Therefore, it is no surprise that it cannot account for the large variance in our data. After all, the highest rated vase in our set has one of the higher *M* scores, which is due to a minimum-to-top ratio of 1:2. In any case, our data show once again that merely counting assumed ideal ratios is not sufficient for predicting the beauty of vases.

The scores of Staudek’s (1999) extended measure *M*_e_ varied to a larger extent across the vases than those of *M* (see Figure 3), which substantially improved the prediction of beauty, although much of the variance still remained unexplained. This indicates that the introduced tolerance margin of 10% around the assumed optimal values is still not sufficient. Rather, our data show that the important variables affect beauty continuously over a large range. Furthermore, which values are optimal seems to depend on the specific conditions. In the present study, we could specifically show this for the MMR. Different from Birkhoff’s assumption, the optimal MMR was not 0.5, but varied depending on the width of the vases from 0.34 to 0.47.

Although the measure *M* was not successful in the present study, Birkhoff’s general idea that geometric relationships are important for the beauty of vases is still valid. This also holds for his assumption that functionality plays an important role. As mentioned, he demanded, among others, that the minimum width should be in the upper half of the vase and at least one-eighth of its height. For our vases this means that their minimum width should be larger than 37.5 px. This criterion was not met by our four vases with an MMR smaller than or equal to 0.21, which also obtained the lowest ratings. The vase with an extremely small minimum of 4.52 px obtained by far the lowest rating. With its rather small neck it seems to be fragile, difficult to grasp., and unsuitable of holding more than one flower.

Given the large effect of the MMR on the beauty ratings, the question arose whether curvature had an effect on the ratings independent of its modulation of the MMR. As our analyses show, this was indeed the case, although the effect was relatively small (about 6% of the variance). The small effect of curvature on the beauty ratings of vases is also reflected in the ratings of the isolated lines. One reason for the small effect of curvature could be its limited range. However, because the outline curvature strongly modulates the minimum width, which should be positive, it could hardly have varied to a greater extent. Consequently, the curvature of the lines did not vary much around that of Hogarth’s Line of Beauty. Nevertheless, the most liked line 3 (see Figure 6) is close to Hogarth’s Line of Beauty. As the comparison with Hogarth’s seven lines revealed, it is most similar to his line 5, which was not significantly less liked than his Line of Beauty (line 4) in the study of Hübner and Ufken (2022). Taken together, our results suggest that a curvature similar to that of Hogarth’s Line of Beauty is also the most preferred when used as a vase outline.

### 3.1. Limitations and future directions

One of the objectives of the present study was to find out whether curvature, in addition to spatial proportions, has an effect on the beauty of the vases. As we have shown, this was indeed the case, at least for vases with an S-shaped contour. Although this result is sufficient for demonstrating that curvature can have an effect, it would still be interesting to investigate in further studies whether this result also holds for vases with other curved shapes, e.g., for those with logarithmically curved contours.

Furthermore, we used young university students as participants in our study. Although it is known that aesthetic preferences vary largely for content and artworks, but little for natural domains and structural object features (Chatterjee, 2004; Vessel et al., 2018), which is partly confirmed by the present study, it would certainly be worthwhile to also investigate the preferences for vases of different shapes with a more heterogeneous sample of participants.

Finally, one of the reviewers noted that the geometric relations are not necessarily the perceived ones. For example, although the base and top width of our vase stimuli are physically identical, they are not always perceived to be the same length. Therefore, empirically measuring the extent of such optical illusions and their contribution to overall beauty is also an interesting question that should be explored in further studies.

## Data availability statement

The datasets presented in this study can be found in online repositories. The names of the repository/repositories and accession number(s) can be found at: https://doi.org/10.17605/OSF.IO/MT2C9.

## Ethics statement

Ethical review and approval was not required for the study on human participants in accordance with the local legislation and institutional requirements. The patients/participants provided their written informed consent to participate in this study.

## Author contributions

RH and EU developed the study concept and design. RH performed the programming, data collection, data analysis, and provided a critical revision. EU computed the aesthetic measures and drafted the manuscript. All authors contributed to the article and approved the submitted version.

## Conflict of interest

The authors declare that the research was conducted in the absence of any commercial or financial relationships that could be construed as a potential conflict of interest.

## Publisher’s note

All claims expressed in this article are solely those of the authors and do not necessarily represent those of their affiliated organizations, or those of the publisher, the editors and the reviewers. Any product that may be evaluated in this article, or claim that may be made by its manufacturer, is not guaranteed or endorsed by the publisher.
